# Treatment outcomes across curve patterns and severities in adolescent idiopathic scoliosis treated with a pattern-specific CAD-CAM brace

**DOI:** 10.3389/fresc.2026.1826976

**Published:** 2026-05-07

**Authors:** Tuğba Kuru Çolak, Burçin Akçay, Hans-Rudolf Weiss, Xiaofeng Nan, Lisa Elliott, Serra Zeynep Akkoyunlu, Maksym Borysov

**Affiliations:** 1 Department of Physiotherapy and Rehabilitation, Faculty of Health Sciences, Marmara University, Istanbul, Türkiye; 2 Department of Physiotherapy and Rehabilitation, Faculty of Health Sciences, Dokuz Eylül University, İzmir, Türkiye; 3 Schroth Best Practice Academy, Neu-Bamberg, Germany; 4 Nan Xiaofeng’s Spinal Orthopedic Workshop, Xi'an Shaanxi, China; 5 ScolioFys Clinic, Copenhagen, Denmark; 6 Department of Biostatistics, Istanbul Faculty of Medicine, Istanbul University, Istanbul, Türkiye; 7 Orttech Plus Rehabilitation Service, Kharkiv, Ukraine

**Keywords:** adolescent, brace, scoliosis, spine, treatment

## Abstract

**Background:**

Whether treatment outcomes differ according to curve pattern or baseline severity in adolescents with idiopathic scoliosis (AIS) remains a subject of debate. In particular, it is unclear whether pattern-specific, CAD-CAM–designed brace systems provide comparable effectiveness across different curvature types. This study aimed to evaluate the influence of curve pattern and initial curve magnitude on treatment outcomes in AIS patients treated with a pattern-specific CAD-CAM–designed brace.

**Methods:**

A retrospective analysis was conducted on female AIS patients aged 10–14 years (Risser 0–2) treated between 2015 and 2024. Cobb angle and angle of trunk rotation (ATR) were used as primary outcome measures. Data from four international clinics were analyzed for changes in spinal curvature and curve pattern.

**Results:**

A total of 145 patients were included (mean age 12.2 years; mean Cobb angle 38.4° ± 11.4°). Post-treatment, mean Cobb angle and ATR values decreased significantly (*p* < 0.001). The overall treatment success rate was 91%, with no significant differences based on apex vertebra location (*p* = 0.459), ALS patterns (*p* = 0.705), or baseline curve severity (*p* = 0.274).

**Conclusion:**

In this multicenter cohort of skeletally immature adolescents with idiopathic scoliosis, pattern-specific brace treatment was associated with significant reductions in both radiographic curvature and trunk rotation. Improvements were observed across different curve patterns and baseline severities. However, given the retrospective design and absence of a comparison group, these findings should be interpreted with caution. Prospective controlled studies are warranted to further validate these observations.

## 1 Introduction

Idiopathic scoliosis is, by definition, a spinal deformity characterized by changes occurring in three planes and along three axes in a healthy child (1). The prevalence of idiopathic scoliosis during adolescence has been reported to be 2.3% (2). The appropriate treatment indications are determined based on the patient's age, Cobb angle, and skeletal maturation. In adolescent idiopathic scoliosis (AIS), conservative treatment options include observation, physical therapy, and brace treatment (3, 4). The effectiveness of brace treatment in reducing curve progression has been demonstrated in the literature (5, 6). According to the guidelines of the International Society on Scoliosis Orthopedic and Rehabilitation Treatment (SOSORT), the most commonly used and recommended brace types for AIS are the Chêneau brace models (7).

The brace used within this study is a Chêneau-type brace, produced through a CAD-CAM system based on the curvature pattern, resulting in an asymmetric model. The goal of this brace is to achieve maximum possible correction while maintaining patient comfort and functionality, using minimal material to enhance wearability (8, 9). Whether such pattern-specific, CAD-CAM–designed brace systems provide comparable outcomes across different curve patterns has not been sufficiently investigated.

To appropriately apply both conservative and surgical treatment techniques, classifications for curvature types are particularly necessary in idiopathic scoliosis (10, 11). Some researchers believe that certain curve types are associated with progression. Several authors have reported that double curves carry a higher risk of progression (12, 13). In contrast, others have indicated that thoracic curves are the most progressive (14, 15). Another study found that thoracolumbar and lumbar curves are somewhat more benign, with a maximum progression rate of approximately 1° per year when the angle reaches 80°–90° in adulthood after skeletal maturity. The thoracic component of double curves showed the least progression (16). However, all of these studies indicated that lumbar curves exhibit the least progression.

In a recent study, the authors reported that Chêneau bracing outcomes were more favorable in patients with main lumbar curves compared to those with main thoracic curves. Additionally, the authors found that thoracic curves exhibited a higher progression risk compared to thoracolumbar/lumbar curves within the same curve pattern (17). Similarly, another study reported a higher failure rate of brace treatment in patients with thoracic curves (18). However, whether brace treatment effectiveness differs according to curve pattern or severity remains insufficiently investigated, particularly in CAD-CAM–based systems. It also remains unclear whether technological advancements in brace design can mitigate previously reported differences in outcomes among curve types.

This study aimed to investigate the effectiveness of pattern-specific brace treatment in adolescent idiopathic scoliosis according to different curvature patterns and baseline curve severity. We hypothesized that treatment outcomes with a CAD-CAM–designed brace would not differ significantly according to curve pattern or initial curve magnitude.

## 2 Materials and methods

This retrospective multicenter study was conducted between 2015 and 2024 in four specialized scoliosis centers located in China, Denmark, Germany and Ukraine. Patients treated with a Gensingen brace and followed for a minimum of 18 months were included in the analysis.

The following inclusion criteria were applied: (1) diagnosis of adolescent idiopathic scoliosis (AIS); (2) female sex; (3) age between 10 and 14 years at the initiation of brace treatment; (4) Risser stage 0–2; and (5) a baseline Cobb angle of ≥20°. Patients with non-idiopathic scoliosis, Risser stages 3–5, neuromuscular, rheumatic, or other orthopedic disorders, prior spinal surgery, concomitant medical conditions that could affect brace treatment, or those receiving any additional scoliosis-specific treatment during the study period were excluded. All consecutive patients meeting the inclusion criteria during the study period were included in the analysis, without additional sampling or selection.

The study was conducted in accordance with the Declaration of Helsinki and the ethics approval for the study was obtained from the Ethics Committee of Bandırma Onyedi Eylül University (2025/102). The study was conducted and reported with reference to the STROBE guidelines.

Patients and their families/caregivers were informed that the treatment outcome data could be used in scientific studies, and their consent was obtained in this regard. Care was taken to ensure that no identifiable information was included when transferring patient data to digital platforms.

Data were documented in a standardized multicenter database, with harmonized data collection and measurement procedures applied across all participating centers. The patient's menarcheal status was recorded in months, while their chronological age at the initiation of treatment was documented in years. Pre-treatment radiographs were evaluated to determine the Risser sign and classify the curvature pattern based on the Augmented Lehnert-Schroth (ALS) classification system (10).

Primary outcome measures included the Cobb angle and the angle of trunk rotation. Brace compliance was assessed based on self-reported daily wear time provided by patients and their parents.

The degree of spinal curvature was quantified using the Cobb method, which entails drawing perpendicular lines to the superior and inferior endplates of the neutral vertebrae on an anteroposterior (AP) radiograph of the entire spine (19) and calculating the angle formed between these perpendiculars. Radiographic evaluations were performed at predefined time points: (1) prior to treatment initiation (baseline), (2) 4–6 weeks after the initial brace application while in-brace, and (3) at the final follow-up, either upon completion of brace treatment or after a minimum of 18 months of treatment. To minimize the influence of the brace on radiographic measurements, all out-of-brace x-rays were obtained at least 24 h after brace removal.

Each radiograph was assessed by experienced clinicians using standardized measurement procedures. Due to the retrospective nature of the study, blinding and formal intra- and inter-rater reliability analyses were not performed. The Cobb angles measured from radiographs taken during the most recent follow-up were analyzed to evaluate treatment outcomes. According to the SOSORT guidelines, treatment outcomes were classified into three categories based on the change in Cobb angle: curve progression (≥ 5° increase), curve stabilization (>−5° and <5° change), and curve correction (≤ −5° decrease) (19).

The Angle of Trunk Rotation (ATR) measurement is the most widely utilized method for the clinical and cosmetic evaluation of scoliosis, recognized for its reliability with a repeatability rate of 86%. Changes in inter-observer ATR measurements are considered clinically significant if they exceed 2° (20). As described by Bunnell, ATR is assessed using a specialized inclinometer, the Scoliometer™. During the Adams forward-bending test, patients are instructed to bend forward with their arms relaxed, and the Scoliometer™ is positioned on the patient's back to measure the maximum rotation of each spinal curve (21). ATR measurements recorded at both baseline and the final follow-up evaluation were analyzed for this study.

Bone maturity, growth velocity, and scoliosis progression risk were evaluated using Risser's sign, a sensitive and reliable method for determining skeletal maturity (22). The Risser sign was assessed on the baseline anteroposterior (AP) radiograph. Ossification of the iliac crest apophysis begins at the lateral edge of the spina iliaca anterior superior, progresses medially, and eventually fuses at the spina iliaca posterior superior. The grading is defined as follows: Grade 0 represents the absence of ossification along the iliac crest apophysis, Grade 1 indicates ossification completion of ≤25%, Grade 2 corresponds to 26%–50% completion, Grade 3 to 51%–75%, and Grade 4 to 76%–100%. Grade 5 is assigned when the epiphyseal plate has fully fused with the ilium (22).

Curve classification was conducted using the Augmented Lehnert-Schroth (ALS) classification, an extension of the original Lehnert-Schroth classification system, encompassing seven distinct curvature types. This classification has been demonstrated in the literature to be a reliable system and is utilized for exercise and brace treatment.

1. 3CH (3-curve with Hip prominence): A functional 3-curve pattern with a prominent hip.
2. 3CTL (3-curve thoracolumbar): A functional 3-curve with hip prominence and a thoracolumbar apex at T12.
3. 3C (3-curve): A more balanced functional 3-curve with a minor and shorter lumbar counter-curve.
4. 3CL (3-curve lumbar): A functional 3-curve pattern with an extended lumbar counter-curve.
5. 4C (4-curve): A functional 4-curve pattern classified as a double major curve.
6. 4CL (4-curve lumbar): A functional 4-curve pattern with a major lumbar curvature.
7. 4CTL (4-curve thoracolumbar): A functional 4-curve pattern with a major thoracolumbar curvature and an apex at L1 (10).

**Figure 1:**
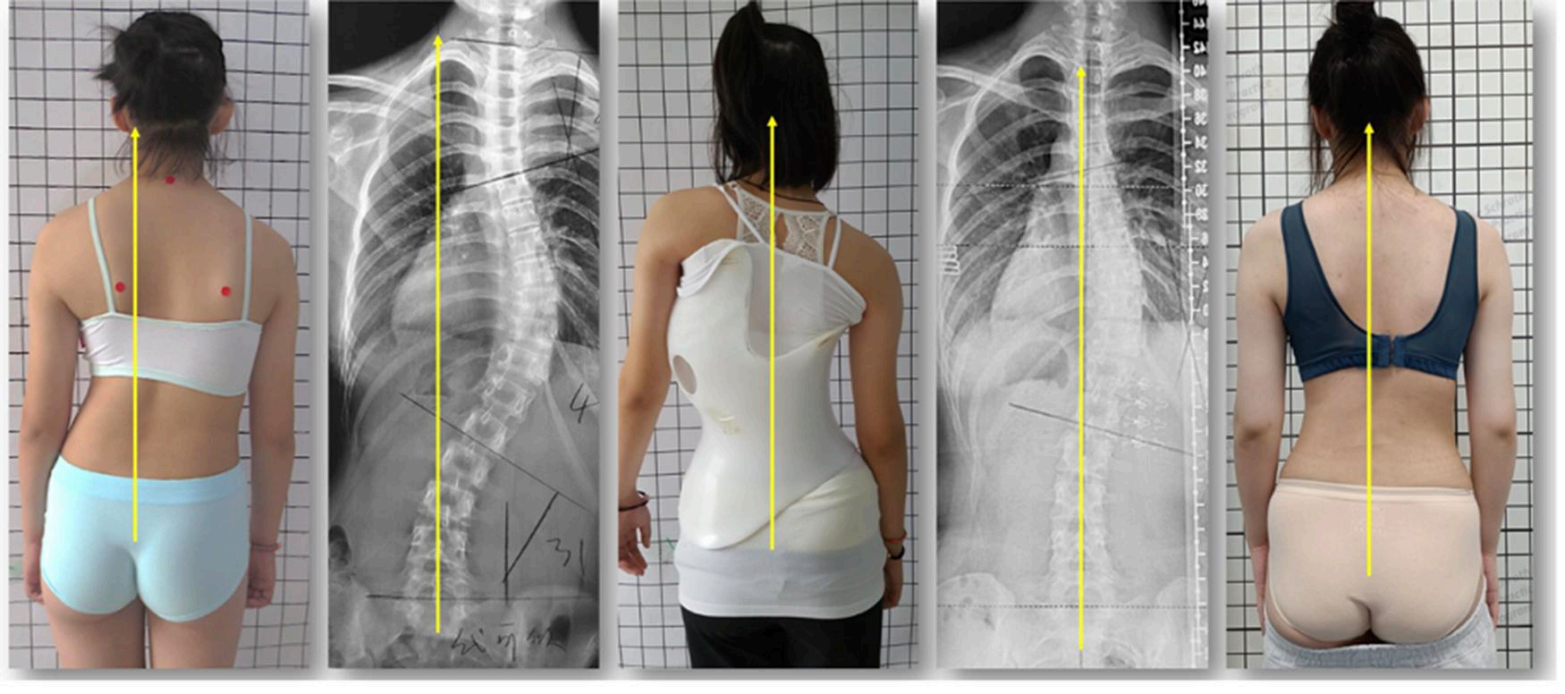
Patient with an AIS (pattern 3CH according to the ALS-classification, born in 2005. In August 2017 the thoracic curvature was 48 degrees. The x-ray taken one year after full weaning of the brace (2022) showed a thoracic curvature of 28 degrees with a marked realignment of the formerly vastly decompensated trunk.

**Figure 2:**
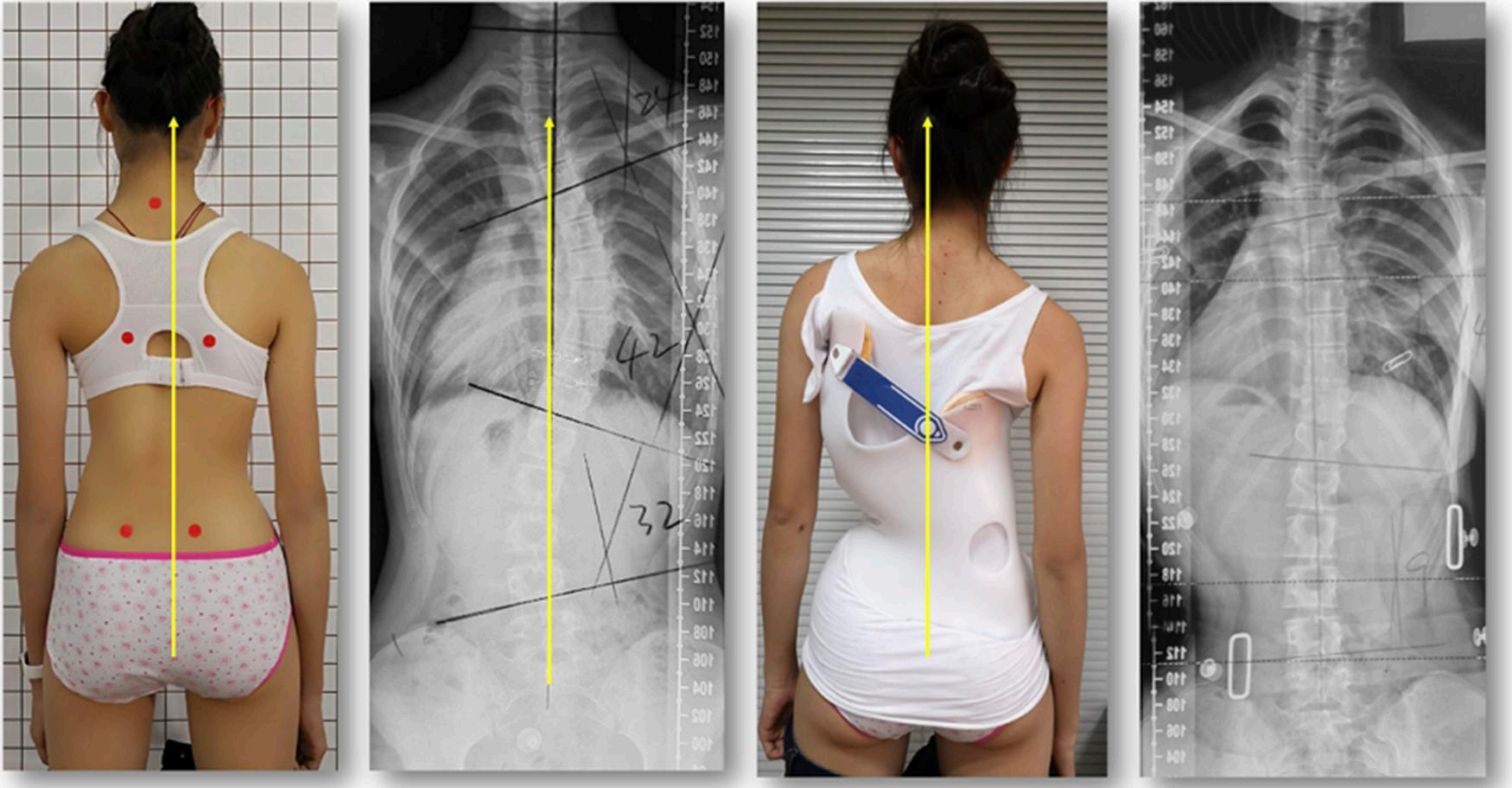
Girl with an AIS, born in 2006 (curve pattern 3CL) according to the ALS-classification treated with a modified 4C version of the brace, showing a reasonable in-brace correction as can be seen on the right.

All participants in this study were treated with a CAD-CAM–based modification of the Chêneau-type brace design, which can be tailored through computer-aided design (CAD). CAD technology enables standardized customization and digital modeling during brace modelling and fabrication. This design allows reduction of brace length, potentially minimizing functional restriction (Figures 1, 2). This approach allows braces to be customized according to individual curvature patterns (8).

In addition to the general CAD-CAM workflow, brace customization was performed through a standardized digital process. The basis for brace adjustment included the patient's clinical record (sex, age, skeletal maturity, Cobb angle, and diagnosis), as well as the geometric characteristics of the deformity. Curve pattern classification was determined using radiographic evaluation and clinical documentation, including photographs of the patient from four directions and standing radiographs.

For brace production, a three-dimensional (3D) scan of the patient's trunk was obtained. Although virtual adjustment can also be performed using manual trunk measurements, a 3D scan was preferred for standardization and practical applicability. The virtual brace adjustment process was performed using commercially available CAD software (Final Surface®) together with customized plug-ins (ScoliCAD®). First, the patient's 3D scan was aligned to a standardized coordinate system to enable derivation of parametric measurements required for initial brace modelling. The scan was then scaled using a semi-automated scaling process (ScoliCAD® Scaler), with additional manual fine adjustments when necessary. Based on the patient's curve pattern and radiographic parameters, an appropriate brace model was selected from a predefined digital library developed according to the ALS classification system. The selected brace model was inserted into the digital environment and adjusted using dedicated adjustment tools (ScoliCAD® Adjuster), followed by manual refinements to ensure appropriate anatomical fit. Using a brace design module (ScoliCAD® Brace Designer), corrective forces were applied and individually increased in the frontal and sagittal planes according to curve characteristics such as apex location, vertebral rotation, and sagittal alignment, with the aim of achieving three-dimensional correction while considering patient comfort.

The finalized brace model represented a negative mold and was exported as a standard STL file for manufacturing. This model was milled from a polyurethane (PU) foam block, which was subsequently used for thermoforming a heated high-density polyethylene (HDPE) sheet. After cooling, the brace was trimmed, finished, and individually adjusted to the patient's body. The PU foam mold was discarded or recycled after production. During follow-up visits, brace adjustments were performed when necessary based on clinical assessment, patient growth, and radiographic findings. All braces were digitally modeled using standardized CAD protocols according to curvature pattern and severity (Cobb angle), allowing patient-specific customization based on individual anatomical and radiographic parameters (8, 9). Detailed technical protocols for brace fabrication and adjustment have been described previously (8, 9).

According to the guidelines (3, 4), patients with Risser stages 0 to 2 and a risk of scoliosis progression exceeding 60%, as determined by the Lonstein and Carlson formula, are recommended to initiate brace treatment. In this study, patients were advised to commence brace treatment following the assessment of their risk of progression. For optimal outcomes, full-time use (min. 20 h. per day) of the brace was recommended (12).

Data analysis was conducted using SPSS version 16 (Statistical Package for the Social Sciences). A *p*-value of <0.05 was considered statistically significant for two-tailed tests. The Kolmogorov–Smirnov test was used to assess the normality of variables. Descriptive statistics, including mean, standard deviation, minimum, and maximum values, were calculated. In double major curvatures (4C), the larger curve, identified as the major curve based on its location, was measured and analyzed for statistical purposes.

As the data did not follow a normal distribution, pre- and post-treatment ATR and Cobb angle measurements were compared using the Wilcoxon signed-rank test. Comparisons of mean Cobb angle and ATR values among patient groups with different curve patterns were performed using the Kruskal–Wallis and Mann–Whitney U tests.

To evaluate potential predictors of treatment response, linear regression analyses were conducted as an exploratory approach to examine associations between changes in Cobb angle and ATR (dependent variables) and curve location, ALS curve pattern, age, and Risser stage (independent variables). Continuous variables were entered directly into the models, while categorical variables (curve location, ALS classification, and menarche status) were included using dummy coding with clinically relevant reference categories. Due to the small sample size of certain subgroups, the 3CTL and 3CH patterns were combined prior to analysis, as these patterns share similar clinical and biomechanical characteristics within the ALS framework.

Model assumptions were evaluated at the residual level, including normality and homoscedasticity. For the ATR change model, where heteroscedasticity was detected, additional analyses using robust (HC3) standard errors were performed, and interpretations were based on these more conservative estimates. Given the exploratory nature of the analysis, no causal inference was intended.

## 3 Results

A total of 145 female AIS patients were included in the study, and their treatment outcomes were analyzed. The mean follow-up period was 34.1 (range: 18–75) months. The average age of the patients was 12.2 ± 1.02 years (range: 10–14), with a mean Cobb angle of 38.4° ± 11.4° (range: 20–81) and a mean ATR angle of 11.9° ± 3.9° (range: 2–22). At baseline, 63 patients (43.4%) had a Cobb angle of ≥40°, whereas 82 patients (56.6%) presented with a Cobb angle below 40°.

The average Risser score was 0.68 ± 0.8 (range: 0–2). Specifically, 84 patients (57.9%) had a Risser score of 0, 23 patients (15.9%) had a score of 1, and 38 patients (26.2%) had a score of 2. Additionally, 95 patients (65.5%) had not yet reached menarche, while the mean post-menarche duration for the 50 patients who had was 6.5 months (range: 1–19).

Based on the location of the apex vertebra, the primary curvature distribution revealed that 104 patients (71.7%) had a primary thoracic curve, 25 patients (17.2%) had a primary lumbar curve, and 16 patients (11%) had a primary thoracolumbar curve. Regarding ALS classification, the most common patterns were 3CH (37 patients, 25.5%) and 4C (35 patients, 24.1%), while the least common pattern was 3CTL, observed in only 2 patients. The average daily brace-wearing time was 20.7 h, with 133 patients (91%) wearing the brace for 18 h or more daily.

**Figure 3:**
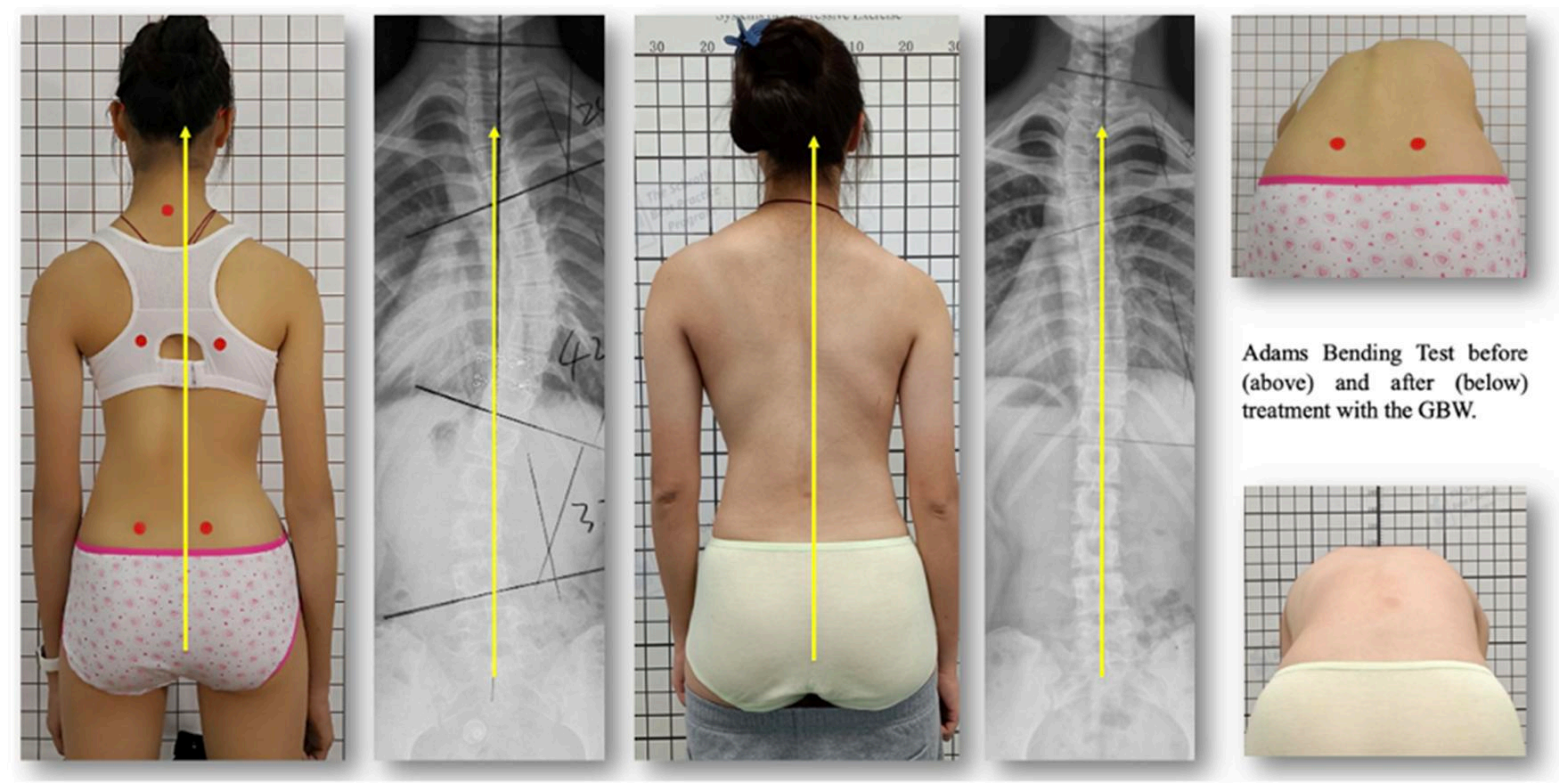
Outcome of the same patient as shown on Figure 2, in 2018, the thoracic curve was 42 degrees, lumbar 32 degrees. Six months after brace weaning (2022), the thoracic curve measured 19 degrees and the lumbar curve 5 degrees. A marked reduction of the rib hump as shown on the right is visible.

**Figure 4:**
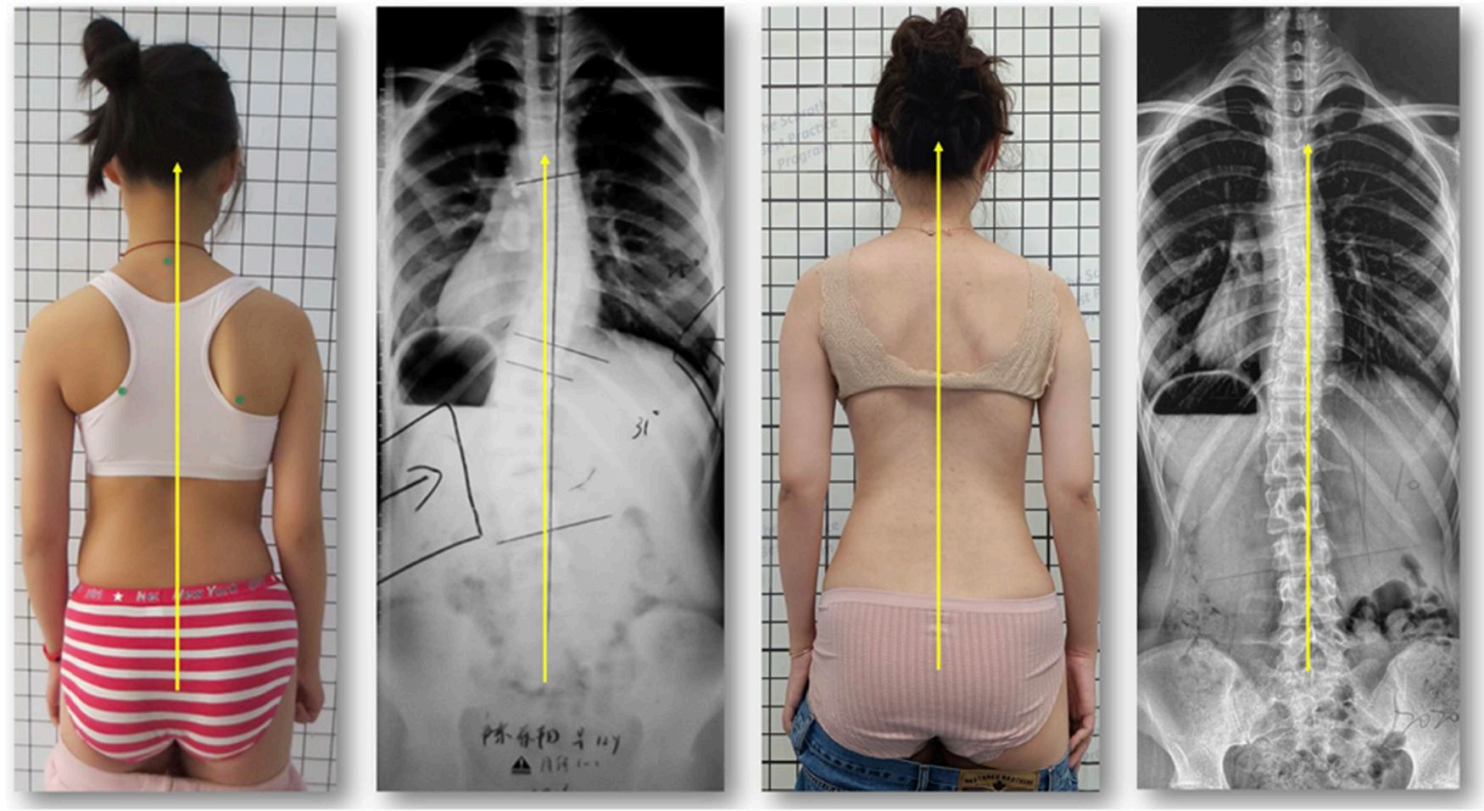
Girl with AIS 4C, born in 2004, in December 2016, the thoracic curvature was 25 degrees, and the lumbar curvature was 31 degrees at the start of treatment with a CAD/CAM chêneau derivative. The x-ray taken one year after full brace weaning (2020) showed a thoracic curvature of 11 degrees and lumbar curvature of 10 degrees. A symmetric trunk shape has been achieved as well.

When the results of all patients were analyzed, the mean maximum Cobb angles and ATR values significantly decreased after treatment (*p* < 0.001) (Figures 1–4). The Cobb angles measured while wearing the brace also showed a statistically significant reduction compared to the initial Cobb angles (*p* < 0.001).

The treatment outcomes showed no significant differences in efficacy among thoracic, thoracolumbar, and lumbar curves based on the location of the apex vertebra (*p* > 0.05, Table 1). Brace treatment demonstrated consistent effectiveness across different curve types in terms of mean changes in Cobb angle and ATR and in-brace Cobb angle values. In the comparison between groups, the differences in the maximum ATR angle were due to the higher ATR values in thoracic curvatures (Table 1).

**Table 1:** Comparison of Cobb angle, ATR values, and changes before and after treatment according to the location of the apex vertebra.

Variables	Main Thoracic *n* = 104 Mea*n* ± SD (Min – Max)	Main Lumbar *n* = 25 Mea*n* ± SD (Min – Max)	Main Thoracolumbar *n* = 16 Mean ± SD (Min – Max)	*p* value
Maximum Cobb angle° before treatment	39.2 ± 11.4 (22–81)	37.5 ± 13.2 (22–71)	33.9 ± 7.9 (20–47)	0.179
Maximum Cobb angle after the treatment	33.8 ± 16.1 (2–86)	30 ± 15.2 (5–63)	27.5 ± 11.6 (11–50)	0.242
Mean difference of Cobb angles	−5.4 ± 9.1 (−26–20)	−7.5 ± 7.6 (−21–6)	−6.3 ± 7.8 (−20–12)	0.634
Cobb in-brace	23.7 ± 13.1 (0–54)	26.1 ± 12.7 (0–51)	23.4 ± 15.3 (5–57)	0.504
Maximum Angle of trunk rotation° before treatment	12.5 ± 3.8 (5–22)	10 ± 3.5 (3–20)	11.3 ± 4.1 (2–20)	0.012*
Maximum Angle of trunk rotation° after the treatment	8.2 ± 4.6 (0–24)	6.6 ± 4.1 (2–15)	5.6 ± 3.2 (2–11)	0.021*
Mean difference of ATR values	−3.3 ± 3.1 (−9–4)	−5.6 ± 3.3 (−12–1)	−4.2 ± 3.9 (−14–4)	0.136

* *p* < 0.05 was considered statistically significant.

When analyzed by ALS patterns, the post-treatment changes in Cobb angle and ATR were similar across the seven curvature patterns (Table 2). In-brace Cobb angle values were also consistent across ALS patterns (*p* = 0.975).

**Table 2:** Comparison of the pre-treatment and post-treatment averages and changes in Cobb angle and ATR values according to ALS pattern.

Variables	Mean ± SD (Min – Max)							
	3CH *n* = 37	3CTL *n* = 2	3CN *n* = 29	3CL *n* = 18	4C *n* = 35	4CTL *n* = 14	4CL *n* = 10	*P* value
Maximum Cobb angle° before treatment	42.1 ± 13 (25–81)	39.5 ± 10.6 (32–47)	37.5 ± 8.8 (27–63)	35.5 ± 10 (24–58)	40 ± 12.7 (22–71)	33.1 ± 7.7 (20–45)	33.6 ± 11.4 (22–60)	0.101
Maximum Cobb angle after the treatment	35.5 ± 18.8 (2–86)	30.5 ± 10.6 (23–38)	33.1 ± 10.8 (15–60)	30.4 ± 13.2 (10–56)	33.6 ± 17.8 (10–81)	27.1 ± 12 (11–50)	27.3 ± 15.4 (5–63)	0.513
Mean difference of Cobb angles	−6.5 ± 10.6 (−26–20)	−9 ± 0 (- 9- - 9)	−4.4 ± 6.9 −20–20	−5.1 ± 7.1 (−17–12)	−6.4 ± 9.2 (−23–17)	−6 ± 8.3 (−20–12)	−6.3 ± 9.3 (−19–6)	0.880
Cobb in-brace	24.8 ± 12.2 (0–51)	27 ± 14.1 (17–37)	23 14.5 (0–54)	24.1 12.3 (6–51)	24.2 13.2 (0–51)	22.9 15.9 (5–57)	24.7 13.6 (0–50)	0.975
Maximum Angle of trunk rotation° before treatment	14.4 ± 3.7 (8–22)	11.5 ± 4.9 (8–15)	12 ± 3.2 (7–19)	10.2 ± 3.4 (5–15)	10.8 ± 3.2 (6–21)	11.3 ± 4.2 (2–20)	10.1 ± 4.8 (3–20)	0.001*
Maximum Angle of trunk rotation° after the treatment	9.6 ± 5.4 (1–24)	3 ± 0 3–3	7.8 ± 3.5 (2–15)	6.8 ± 3.8 (0–14)	7.5 ± 4.2 (0–18)	6.1 ± 3.2 (2–11)	5.4 ± 4.1 (2–15)	0.017*
Mean difference of ATR values	−4.8 ± 3.8 (−14–2)	−8.5 ± 4.9 (−12 – −5)	−4.1 ± 3 (−12–3)	−3.4 ± 4.5 (−14–3)	−3.3 ± 3.9 (−13–4)	−5.2 ± 3.1 (−9–1)	−4.7 ± 3.1 (−9–0)	0.250

* *p* < 0.05 was considered statistically significant.

Regarding curve severity, the brace showed comparable effectiveness in reducing Cobb angle and ATR for curves below and above 40° (*p* > 0.05, Table 3). In-brace Cobb angle values were similar for moderate and severe curves (*p* = 0.796).

**Table 3:** Comparison of the pre-treatment and post-treatment averages and changes in Cobb angle and ATR values according to curve severity.

Variables	Cobb angle < 40° *n* = 82 Mean ± SD (Min – Max)	Cobb angle ≥ 40° *n* = 63 Mean ± SD (Min – Max)	*P* value
Maximum Cobb angle° before treatment	30.2 ± 4.5 (20–39)	48.9 ± 8.9 (40–81)	<0.001*
Maximum Cobb angle after the treatment	23.9 ± 9.5 (2–54)	43.7 ± 14.9 (19–86)	<0.001*
Mean difference of Cobb angles	−6.3 ± 8.2 (−26–20)	−5.1 9.3 (−23–20)	0.383
Cobb in-brace	23.2 ± 12.6 (0–50)	24.7 13.7 (0–57)	0.796
Maximum Angle of trunk rotation° before treatment	10.4 ± 3.4 (2–20)	13.9 ± 3.6 (6–20)	<0.001*
Maximum Angle of trunk rotation° after the treatment	5.9 ± 3.4 (0–15)	10 ± 4.6 (1–24)	<0.001*
Mean difference of ATR values	−4.5 ± 3.3 (- 14–3)	−3.9 ± 4.2 (−14–4)	0.365

* *p* < 0.05 was considered statistically significant.

According to the predefined outcome criteria, 9% of patients demonstrated curve progression (≥5° increase). When correction (≥5° decrease) and stabilization (>−5° and <5° change) were considered together, the overall treatment success rate was 91% across all curvature patterns.

Although the reduction in curve magnitude was slightly greater in thoracolumbar curves, treatment success rates were statistically similar among thoracic, thoracolumbar, and lumbar curves (*p* = 0.459, Table 4). Similarly, when analyzed according to ALS classification, a greater proportion of cases with curve reduction was observed in the 3CTL and 4CTL patterns; however, overall treatment success rates did not differ significantly among ALS curve types (*p* = 0.705). Baseline curve severity (<40° vs. ≥40°) also did not significantly influence treatment success (*p* = 0.274).

**Table 4:** Evaluation of curvature correction status based on the change in the Cobb angle.

Variables	Changes in Maximum Cobb angle°	Curve correction (≤−5)	Curve stabilization (>−5 and <5°)	Curve progression (≥ 5 )
According to the location of the apex vertebra	Main Thoracic	56 (53.8%)	37 (35.6%)	11 (10.6%)
	Main Lumbar	14 (56%)	10 (40%)	1 (4%)
	Main Thoracolumbar	12 (75%)	3 (18.8%)	1 (6.2%)
*p* value	0.459			
According to ALS Classification	3CH	23 (62.2%)	9 (24.3%)	5 (13.5%)
	3CTL	2 (100%)	-	
	3CN	13 (44.8%)	14 (48.3%)	2 (6.9%)
	3CL	10 (55.6%)	7 (38.9%)	1 (5.6%)
	4C	20 (57.1%)	12 (34.3%)	3 (8.6%)
	4CTL	10 (71.4%)	3 (21.4%)	1 (7.1%)
	4CL	4 (40%)	5 (50%)	1 (10%)
*p* value	0.705			
According to the curve severity	Cobb angle < 40°	51 (62.2%)	24 (24.3%)	7 (8.5%)
	Cobb angle ≥ 40°	31 (49.2%)	26 (41.3%)	6 (9.5%)
*p* value	0.274			

In univariable linear regression analyses, Risser stage was not associated with Cobb change (R^2^ = 0.000011, *p* = 0.968) or ATR change (R^2^ = 0.000065, *p* = 0.923). In the multivariable exploratory model for Cobb change, only baseline Cobb angle remained significantly associated with the outcome (*β* = 0.174, *p* = 0.010), while the overall model was not statistically significant (R^2^ = 0.090, *p* = 0.461). In the ATR change model, baseline ATR was the only robust independent predictor (*β* = −0.302, *p* = 0.002, based on HC3 robust standard errors). No significant association was observed for Risser stage in any model. These findings should be interpreted cautiously due to the exploratory nature of the analyses and subgroup size limitations.

## 4 Discussion

The present study demonstrated that brace treatment resulted in a 91% success rate in female adolescents with idiopathic scoliosis during their rapid growth phase. Treatment success was comparable across different curve patterns and was similarly high in curves exceeding 40° and those below 40° at baseline.

Curve classification systems play a central role in the conservative management of scoliosis by guiding exercise protocols, brace design, and surgical decision-making (10, 23). In adolescent idiopathic scoliosis (AIS), more than 90% of curves follow recognizable structural patterns, whereas non-idiopathic scoliosis typically presents with atypical configurations (23). The Lehnert-Schroth classification was originally developed in the 1970s for exercise-based treatment and later adapted for Chêneau-type bracing (23). Subsequently, the Augmented Lehnert-Schroth (ALS) classification was later adapted for use in conservative scoliosis management, including exercise- and brace-based approaches (10, 23). Despite the widespread clinical use of these classifications, relatively few studies have examined brace effectiveness according to conservative-treatment-based pattern systems. Therefore, the present study investigated treatment outcomes according to both apex vertebra location and ALS curve classification.

Previous studies evaluating the natural history of AIS and brace treatment outcomes have suggested that prognosis may differ according to curve type. Double curves and thoracic curves have been reported to show greater progression, whereas thoracolumbar and lumbar curves tend to progress more slowly (10–14). Similarly, several brace studies have reported higher correction rates in lumbar and thoracolumbar curves compared with thoracic curves (15, 16, 24–27). For example, a study of 177 AIS patients treated with a Chêneau brace and classified according to the modified Lenke system demonstrated greater curve reduction in mLenke V and VI patients, with lumbar curves showing superior in-brace correction compared to thoracic curves (15). Weiss et al. reported correction rates of 62% in lumbar/thoracolumbar patterns, 36% in thoracic curves, and 50% in double major curves using the Chêneau light brace (25). In another study examining the effect of thoracolumbosacral orthosis (TLSO) on curve types in 168 patients, the rate of surgery or progression was reported as 30.3% for main thoracic curves and 5.3% for main lumbar curves in patients compliant with brace use. The researchers reported that the highest progression was observed in mLenke II curves at 54.5%, while the lowest was in mLenke V curves at 13.6% (16). However, the term TLSO is a very broad definition, and the exact model of the brace used in this study was not specified. The authors stated that either a Boston brace or some not furtherly specified computer-aided design brace was used (16).

Cheung and colleagues, in their study involving regression analysis, reported that AIS patients prescribed underarm braces with major thoracic curves had a higher risk of progression compared to those with major lumbar curves (27).

Ohrt-Nissen and colleagues emphasized in their study, which compared supine lateral bending radiographs with in-brace radiographs of patients using the Providence brace, that the corrective effect of the brace was similar across different curve types (28). In a recent analysis, in-brace correction was shown to vary substantially by curve type, with thoracic curves demonstrating an average correction of 46%, compared with 73% in thoracolumbar curves (29).

The same study also found that skeletally immature patients (Risser 0) treated with a Boston-style TLSO had approximately a 5.6-fold higher likelihood of requiring surgery compared with those treated with a Rigo Chêneau or Providence brace (29).

In contrast to several previous reports suggesting that brace effectiveness varies by curve type, the present study did not demonstrate statistically significant differences in treatment success between groups. Although thoracolumbar curves (3CTL and 4CTL) demonstrated numerically higher correction rates, these differences did not reach statistical significance. These findings are consistent with previous research evaluating Schroth Best Practice exercises combined with Chêneau-type bracing, in which long-term outcomes did not significantly differ according to curve pattern (30). Consistent with prior literature, progression was most frequently observed in main thoracic and 3CH patterns; however, no statistically significant differences in overall success rates were observed between groups.

Thoracic curves are generally considered less flexible due to the structural influence of the rib cage (31), which may limit brace-induced correction. Nevertheless, our results suggest that meaningful correction and progression control can be achieved even in thoracic curves when treated with a pattern-specific CAD-CAM–designed brace during the rapid growth phase.

Baseline curve magnitude has been identified as a risk factor for progression (26), yet evidence regarding brace treatment in curves exceeding 40° remains limited. Previous studies reported success rates of 92% in patients with a mean Cobb angle of 44.2° treated with the Gensingen brace (32) and 91% in patients with a mean Cobb angle of 47° treated with the Progressive Action Short Brace or Lyon brace (33). In the present study, patients with a mean initial Cobb angle of 48.9° achieved a 90.5% success rate, which was comparable to the 91.5% success observed in patients with curves below 40°. These findings suggest that high initial curve magnitude may not necessarily preclude favorable outcomes when treatment is initiated during the rapid growth period.

As already noted, the success rate (proportion of stabilized and improved curvatures) of braces currently available for the treatment of scoliosis varies considerably (6). Success rates of between less than 50% and more than 90% have been reported, particularly for braces known as Chêneau derivatives, which are designed to initiate a more or less pronounced corrective movement, while less asymmetrical, more compressive braces achieve relatively stable success rates of around 70% (6).

In a more recent analysis, the reasons given for this were the increased potential for error with asymmetrical braces, while compressive braces tend to have a more non-specific effect (34).

Unfortunately, most publications on this topic today only publish the names of the braces, but not exact descriptions, let alone pictorial representations of the orthoses used in the individual publications (5, 35, 36). Therefore, it is not possible for us to discuss the different designs of the various orthoses used in other studies in a comparative analysis, which may underlie the deviations from our results described above.

In view of the sometimes, considerable restrictions on quality of life caused by many corrective orthoses, success rates of less than 70% are unacceptable. Quality assurance measures in scoliosis treatment with orthoses are therefore required (37). To the best of our knowledge, no studies have investigated how ATR changes in relation to curve patterns during brace treatment. Therefore, it was not possible to compare our results. Kinel et al. reported that in children with an average Cobb angle of 55° who had been undergoing brace treatment for six months, the maximum ATR values were significantly lower compared to children with an average Cobb angle of 59° who did not receive brace treatment (37). In our study, the mean ATR value decreased by −4.5 in patients with an average Cobb angle below 40° and by −3.9 in those with an average Cobb angle above 40°. However, when changes in ATR values were compared according to curve severity and pattern, the groups were found to be similar in terms of ATR improvement.

In the literature, studies investigating the effectiveness of brace treatment according to AIS curve patterns remain limited. In this context, the present study contributes to the existing body of knowledge by evaluating treatment outcomes across both curve patterns and severities. The inclusion of a homogeneous high-risk population (female patients with Risser 0–2 during the rapid growth phase), as well as the assessment of both moderate and severe curves, can be considered strengths of this study. Deformities may be perceived differently by patients depending on curve pattern; however, patient-reported outcomes and expectations regarding brace treatment were not evaluated, which represents a limitation. The minimum follow-up duration of 18 months may also be considered a limitation. Nevertheless, this period corresponds to the phase of highest risk for curve progression in skeletally immature patients (35). According to Lonstein and Carlson (14), patients in this phase present a high risk of progression, emphasizing the clinical relevance of early treatment outcomes. However, longer-term follow-up is necessary to determine the durability of correction after skeletal maturity. Previous reports have suggested that correction achieved during treatment may regress within two years after brace discontinuation (38), whereas more recent studies indicate that long-term deterioration may be limited with contemporary brace designs. For example, Aulisa et al. reported sustained correction more than 10 years after brace weaning using an asymmetric high-correction orthosis (39).

The strengths of this study include the inclusion of a homogeneous high-risk population (female patients, Risser 0–2, rapid growth phase) and the evaluation of both moderate and severe curves. However, several limitations should be acknowledged. Patient-reported outcomes and satisfaction were not assessed, and brace compliance was not monitored using objective measurement tools (e.g., temperature or sensor-based wear-time monitors). The minimum follow-up duration of 18 months may be considered a limitation of the present study. However, this period corresponds to the phase of highest risk for curve progression in skeletally immature patients. Therefore, the observed treatment effects likely reflect clinically meaningful outcomes during the most critical stage of growth. Nevertheless, longer-term follow-up is required to evaluate the durability and stability of correction after skeletal maturity. Future prospective studies with extended follow-up should also compare different brace models across curve patterns and incorporate patient-centered outcomes to better understand both radiological and clinical effectiveness.

Potential sources of bias, including the retrospective design and the use of self-reported brace adherence, should be considered when interpreting the findings.

## 5 Conclusions

This study indicates that pattern-specific CAD-CAM–designed bracing was associated with favorable radiographic and clinical outcomes in adolescents with idiopathic scoliosis during the rapid growth phase. Improvements in Cobb angle and ATR were observed across different curve patterns and severities, without statistically significant differences between groups.

However, given the retrospective design and the absence of a comparison group, these findings should be interpreted with caution. Further prospective controlled studies are needed to better understand the effectiveness of this approach across different patient subgroups.

## Data Availability

The raw data supporting the conclusions of this article will be made available by the authors, without undue reservation.
